# Hysterical Epilepsy

**Published:** 1844-12

**Authors:** 


*Hysterical Epilepsy.*—A girl, aged sixteen years, had menstruated during the last year, once a fortnight, with leucorrhœa intervening. Three weeks before admission had a fit, apparently epileptic, preceded by sickness after eating. The fit was sudden, and she remained unconscious for more than an hour, in an out-house, and unseen; this was followed by headache and pains in her limbs. She had a second attack, both at the approach of the catamenia. On admission, the heart's action was very irritable, pulse 100. Thoracic and abdominal viscera sound. Sulphate of zinc was administered, at first in doses of two grains thrice a day, increased to eight grains ter die, without sickness or other inconvenience. She was soon discharged cured.—*Med. Chir. Review.*

